# Transcriptome Dynamics in *Triticum aestivum* Genotypes Associated with Resistance against the Wheat Dwarf Virus

**DOI:** 10.3390/v15030689

**Published:** 2023-03-06

**Authors:** Abdoallah Sharaf, Przemysław Nuc, Jan Ripl, Glenda Alquicer, Emad Ibrahim, Xifeng Wang, Midatharahally N. Maruthi, Jiban Kumar Kundu

**Affiliations:** 1 Plant Virus and Vector Interactions, Centre for Plant Virus Research, Crop Research Institute, 16106 Prague, Czech Republic; abdoallah.sharaf@umbr.cas.cz (A.S.); przemyslaw.nuc@amu.edu.pl (P.N.); ripl@vurv.cz (J.R.); glenda.alquicer@vurv.cz (G.A.); emad.ibrahim@vurv.cz (E.I.); 2 State Key Laboratory for Biology of Plant Diseases and Insect Pests, Institute of Plant Protection, Chinese Academy of Agricultural Sciences, Beijing 100081, China; wangxifeng@caas.cn; 3 Agriculture, Health and Environment Department, Natural Resources Institute, Medway Campus, University of Greenwich, Chatham, Kent ME4 4TB, UK; m.n.maruthi@gre.ac.uk

**Keywords:** WDV, wheat, resistance, RNA-seq, transcriptome, genotype, virus

## Abstract

Wheat dwarf virus (WDV) is one of the most important pathogens of cereal crops worldwide. To understand the molecular mechanism of resistance, here we investigated the comparative transcriptome of wheat genotypes with different levels of resistance (Svitava and Fengyou 3) and susceptibility (Akteur) to WDV. We found a significantly higher number of differentially expressed transcripts (DETs) in the susceptible genotype than in the resistant one (e.g., Svitava). The number of downregulated transcripts was also higher in the susceptible genotype than in the resistant one (Svitava) and the opposite was true for the upregulated transcripts. Further functional analysis of gene ontology (GO) enrichment identified a total of 114 GO terms for the DETs. Of these, 64 biological processes, 28 cellular components and 22 molecular function GO terms were significantly enriched. A few of these genes appear to have a specific expression pattern related to resistance or susceptibility to WDV infection. Validation of the expression pattern by RT-qPCR showed that *glycosyltransferase* was significantly downregulated in the susceptible genotype compared to the resistant genotypes after WDV infection, while *CYCLIN-T1-3*, a regulator of *CDK* kinases (cyclin-dependent kinase), was upregulated. On the other hand, the expression pattern of the transcription factor (TF) *MYB* (TraesCS4B02G174600.2; myeloblastosis domain of transcription factor) was downregulated by WDV infection in the resistant genotypes compared to the susceptible genotype, while a large number of TFs belonging to 54 TF families were differentially expressed due to WDV infection. In addition, two transcripts (TraesCS7A02G341400.1 and TraesCS3B02G239900.1) were upregulated with uncharacterised proteins involved in transport and regulation of cell growth, respectively. Altogether, our findings showed a clear gene expression profile associated with resistance or susceptibility of wheat to WDV. In future studies, we will explore the regulatory network within the same experiment context. This knowledge will broaden not only the future for the development of virus-resistant wheat genotypes but also the future of genetic improvement of cereals for resilience and WDV-resistance breeding.

## 1. Introduction

Wheat dwarf virus (WDV), genus *Mastervirus* family *Geminiviridae*, is an important viral pathogen of cereal crops worldwide. The virus was first discovered in wheat plants in the former Czechoslovakia [1]. WDV is transmitted by all instars of the leafhopper species *Psammotettix alienus* in a circulative, nonpropagative manner [2]. WDV has a single-stranded circular DNA genome of about 2.7 kb, which is divided into several functional regions: a short intergenic region (SIR) containing the origin of replication and sites with potential promoters for viral genes grouped into two open reading frames for viral movement (MP) and the coat protein (CP); a long intergenic region (LTR); and a complementary open reading frame that produces two additional viral proteins—the replication protein (Rep) and the replication-associated protein (RepA)—by differential splicing [3]. The Rep protein is responsible for replicating viral DNA, and the RepA is responsible for promoting the production of coat proteins and movement proteins, as well as interactions with plant cell cycle mechanisms [4]. The coat protein is the only viral protein present in the virions and is responsible for circulation of the virus within the insect vector and intracellular movement. The movement protein is required for cell-to-cell movement of the virus [5].

WDV causes dwarfing disease in small-grain cereals, which is manifested by dwarfing, mottling, streaking of the stems, reduction in root size, severe stunting, and decline of infected hosts with up to 90% yield loss [6,7,8,9,10,11,12]. Since its first discovery, WDV has been gradually detected in various wheat-growing areas worldwide and has become a serious problem for cereal growers [13]. Although the number of cereal genotypes showing resistance/tolerance to wheat dwarf disease (WDD) is limited, a few wheat genotypes show some degree of resistance to WDV [11,14,15,16], but little is known about the nature and genes involved in these sources of natural resistance with the exception of a few promising quantitative trait loci (QTL) identified in wheat [17]. Resistance in these genotypes is usually characterised by mild disease symptoms and low virus titre in plants, indicating an active defence response [18]. Naturally, plants have developed specific defence responses in host cells to combat virus (pathogen) attacks mediated by different signalling pathways [19,20]. These are often triggered by host–pathogen recognition and interaction and result in host susceptibility or resistance, which depends on the combination of virus–host genotype and pathogenicity determinants of the virus that recognise and interact with host-specific proteins encoded by *R* genes [21,22]. In addition to the *R*-genes, the interaction between plants and viruses appears to be even more complex, involving various signal transduction pathways that may confer plant defence via inducible mechanism of resistance [22,23,24].

The RNA sequencing (RNA-seq) technology has become a very popular tool for studying multiple interactions in biotic and abiotic stresses [24,25]. RNA-seq offers a complex view of comparative gene expression to elucidate the basal defence response during plant-virus interactions [26,27]. The profile of transcriptome and the target gene expression due to virus infection are not obviously unique and are limited to specific host–virus interactions [26]. In recent years, the comparative analysis of transcriptome has been widely used to determine the expression profile of genes associated with resistance or susceptibility of plant genotypes for several viruses [28,29,30,31,32,33]. In this study, we analysed transcriptome in three wheat genotypes with different levels of resistance to WDV in order to identify and correlate the differential expression of RNA transcripts of the genes that may be associated with resistance. The analysis revealed modulation of several genes associated with susceptibility and resistance to the wheat genotypes. Our results also suggested the importance of different metabolic and signalling pathways in gene regulation and wheat resistance to WDV.

## 2. Materials and Methods

### 2.1. Plant Growth and WDV Inoculation

Eleven seeds of each genotype studied were sown individually in 0.23 L plastic pots filled with a soil mixture (60% luvic chernozem and 40% arenic regosol). Before sowing, 0.9 g of NPK (12.4% N, 11.4% P_2_O_5_, 18% K_2_O) and 0.7 g calcium nitrate (15.5% N) were individually added to soil for every plant. The plants were grown in a greenhouse at 15 °C (for 14 h of light) and at 7.0 °C (for 10 h of darkness) until the samples were collected. Wheat plants of three genotypes, Akteur, Fengyou 3 and Svitava were isolated using a small insect isolator cage and individually inoculated using three viruliferous leafhoppers. Inoculation feeding started at growth stage BBCH 12-13/21 (the 19th day of the plants’ cultivation). *P. alienus* individuals and the WDV wheat strain [34] (accession number: FJ546188) were obtained from the virus collection maintained at the Crop Research Institute, Prague. Inoculation feeding lasted for 8 days; after which, the leafhoppers and isolators were removed. In the period of clearly visible WDV symptoms and differences in reaction between genotypes to WDV infection (the 99th day of plants cultivation), plants were removed from the soil and their roots were carefully washed. Any surface water was removed using a paper towel and plants were weighed in a fresh state. The collected samples of whole plants were kept in a freezer (−80 °C) until laboratory evaluation.

### 2.2. Resistance Assessment of Wheat Genotypes to WDV

WDV titres were assayed by qPCR as previously described by Gadiou et al. [35]. The absolute quantification of viral DNA copies was performed by using a LightCycler^®^ 480 (Roche, Basel, Switzerland). The PCR Master Mix comprises 6 μL of LightCycler^®^ 480 SYBR Green I Master Mix (Roche, Basel, Switzerland) and 0.6 μL of the primer pair mix (UnivWDVfw, UnivWDVrv in final concentration of 10 µM). The thermal cycling protocol included initial denaturation at 95 °C for 10 min, followed by 40 cycles of 95 °C for 15 s, 60 °C for 1 min, 95 °C for 15 s and 60 °C for 15 s. The fluorescence was measured via a 60–97 °C melting curve. The WDV titre in each sample was calculated with a standard curve that was prepared by cloning the target viral DNA into the vector pGEM-T Easy Vector (Promega, Madison, Wisconsin, USA,). The following mathematical formula was applied: number of copies = (amount of DNA × 6.022 × 10^23^)/(length of the plasmid × 1 × 10^9^ × 660) [36] by using the Avogadro’s constant of 6.023 × 10^23^ mol^–1^. All the samples with WDV infection and standards detected by qPCR were measured in triplicates. Following Levesque-Sergerie et al. [37], we defined the detection limits of our qPCR as follows: Cts < 22 are strong positive reactions indicating abundant target nucleic acid in the sample; Cts from 23–29 are positive reactions indicating minimal amounts of target nucleic acid; and Cts > 30 are weak reactions that could represent environmental contamination. This is the range of Ct values for the NTC (non-template control). These values can be considered as zero in the calculations. The Ct values were determined with LightCycler 480 using the fit points method and then processed in Excel to calculate copy numbers and in GraphPad Prism9 for linear regression (calibration curve) and two-way response ANOVA.

### 2.3. RNA Isolation, cDNA Library Construction and Sequencing

A slightly modified Trizol [38] method was used to extract total RNA from 18 individual samples of the three genotypes derived from three biological replications of each genotype (both WDV inoculated and non-inoculated controls). Immediately prior to use, 100 µL of 10% *w*/*v* sodium lauryl sarcosine and 100 µL of 0.5 M EDTA were added to a freshly prepared Trizol mixture. Total RNA was precipitated with isopropanol and re-precipitated with sodium acetate (0.3 M) and ethanol (2.5 vol) to give similar A260/A230 and A260/A280 ratios in all samples. RNA quality was checked by agarose gel electrophoresis of 3 µg of total RNA isolated from each sample. Next, the RNA samples were diluted and measured RIN on the Agilent Bioanalyzer 6000 system (Santa Clara, CA, USA) using the RNA6000 nano-chip. RIN of all RNA samples was in the range of 8.3–9.2, 100 ng of total. Approximately 100 ng of the extracted RNA was used for complementary DNA (cDNA) library construction and subsequent Illumina sequencing. Eighteen cDNA libraries were generated using QuantSeq 3′mRNA-Seq Library Prep Kit for Illumina (Lexogen, Vienna, Austria) following the manufacturer’s instructions. The final cDNA library was created based on PCR amplification, and library quality was assessed on the Agilent Bioanalyzer 6000 system (Santa Clara, CA, USA) with 2100 expert High Sensitivity DNA Assay. The resulting cDNA library was sequenced using the Illumina NextSeq 550 platform (San Diego, CA, USA) following the manufacturer’s recommendations at the Laboratory of Genomics and Bioinformatics, Institute of Molecular Genetics of the Czech Academy of Sciences. 

### 2.4. Transcriptome Analysis

First, quality-control filtering was performed by removing reads containing adapter or poly-N, the low-quality bases were determined with the FastQC program, and high-quality clean reads were obtained. All the subsequent analyses were conducted using high-quality clean reads. The resulting clean reads were aligned to the reference genome (IWGSC) using the Bowtie2 v2.4.4 software (http://bowtie-bio.sourceforge.net/bowtie2/index.shtml, accessed on 5 March 2021) as previously described [39]. In addition, the annotated transcripts were then used for further analyses as described below.

### 2.5. Differential Expression Analysis of mRNAs

The transcript abundances of mRNAs were quantified using the normalised expression values as fragments per kilobase per million reads (FPKM) by the Cuffdiff v2.2.1 software [40]. Differentially expressed transcripts (DETs) between WDV-infected and non-inoculated genotypes were analysed using the edgaR and DESeq2 R packages [41,42]. The expression-based sample clustering and the principal component analysis (PCA) were performed using the R package (v3.24.3) (https://www.r-project.org, accessed on 5 March 2021). Furthermore, genotypes specific DEmRNAs were used for statistical enrichment testing and gene ontology (GO) analysis using PANTHER tool v. 16 [43]. Finally, the Kyoto Encyclopedia of Genes and Genomes (KEGG) was used to analyse the complex biological process of DETs [44]. We obtained all the pathway items in which all the genotypes-DETs were involved, then global metabolism, stress- and infections-related metabolic pathways were presented. Moreover, the number of DETs and its fold of changes for each KEGG pathway were also calculated.

### 2.6. RT-qPCR Analysis

The quantitative reverse transcription PCR (RT-qPCR) analysis was performed using three biological and three technical replicates of total RNA. First strand cDNA fragments were synthesised using 1 µg of total RNA, RevertAid Reverse Transcriptase 200 U/µL and oligo (dT)_18_ (Thermo Scientific, Waltham, MA, USA) according to the manufacturer’s instructions. The specific primers for RT-qPCR were designed using NCBI Primer-BLAST (RRID:SCR_003095) (Supplementary Tables S1–S6). Melting temperatures between 59–61 °C and amplicon length ranging from 70 to 180 bp were the restrictive parameters for primer selection. Other parameters were kept at the default setting. Allowing only a maximum of two mismatches between the primer and the target sequence, we carefully checked the position in the primer sequence and avoided mismatches, especially in the last five nucleotides of the 3′ end. The final oligos were then purchased from Eurofins Genomics (Ebersberg, Germany). A cDNA sample from a pool of equivalent quantities of each treatment condition was prepared for primer validation. Firstly, a thermal gradient PCR was run with a 20 µL reaction containing 1 ul of the 1:20 diluted pool-cDNA sample, 10 µM forward and reverse primers, nuclease free water and DreamTaq Green PCR Master Mix (Thermo Scientific, Waltham, MA, USA). Cycles were set as recommended by the manufacturer. The amplicons visualised in a 1% agarose gel showed primer specificity, purity and correct size at a 60 °C optimal annealing temperature. After this, the reaction efficiency was calculated for each pair of primers performing a six-point standard curve from the pooled cDNA sample and with four-fold serial dilution of the cDNA. Amplifications were performed with a LightCycler 480 Instrument II (Roche, Basel, Switzerland) in 384-well plates containing 10 μL reaction solutions per well using LightCycler^®^ 480 SYBR Green I Master mix (2× concentrated) to which the corresponding forward and reverse primers were added (10 µM and 4 μL of cDNA template. This process was performed once for each wheat genotype. The cycling conditions were 95 °C for 10 min followed by 40 cycles of 95 °C for 5 s and 60 °C for 30 s and 72 °C for 10 s. The resulting Ct values were used to calculate the efficiency from the given slope, after running the standard curves, following the formula E (%) = (−1/(10^slope^ – 1)) × 100. Considering 100% = 2, an acceptable range is between 1.8 to 2.2 [45].

RT-qPCR was performed on the LightCycler 480 system (Roche, Basel, Switzerland) using a LightCycler^®^ 480 SYBR Green I Master mix (Roche, Basel, Switzerland) in 12 μL reactions. The PCR was run at 94 °C for 10 min, 45 cycles of 94 °C for 10 s, 60 °C for 10 s and 72 °C for 10 s. The fluorescence was measured via a 60–97 °C melting curve. The relative expression level of the selected genes to the internal control genes was calculated using the ratio = 2^−ΔΔCT^ [46]. Two reference genes TubB and GAPDH [47] were utilised in this study.

## 3. Results

### 3.1. Evaluation Resistance of Wheat Genotypes to WDV

**Figure 1:**
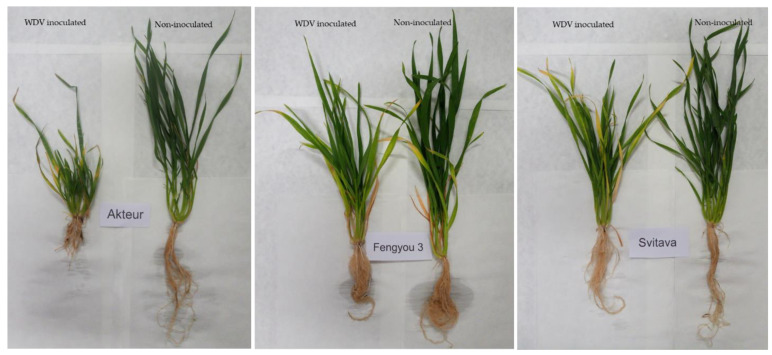
Disease symptoms (80 days post-inoculation) in each tested wheat genotypes (WDV inoculated and non-inoculated control).

The resistance of the wheat genotypes in this study was assessed by observing the disease symptoms after virus infection and by analysing the virus titre using qPCR. The highly resistance/susceptibility of the Svitava/Akteur genotypes was known from our previous study [16] and was investigated in the case of the Chinese genotype Fengyou 3, which also performed well in the field after virus inoculation. The Svitava and Fengyou 3 genotypes showed mild disease symptoms after WDV inoculation, such as slight leaf yellowing, little dwarfing and only a slight reduction of the root system compared to the non-inoculated control. On the other hand, the genotype Akteur showed very severe disease symptoms associated with severe yellowing and dwarfing and a severe reduction of the root system. These results show good resistance of Svitava and Fengyou 3, as well as high susceptibility of Akteur to WDV (Figure 1).

**Figure 2:**
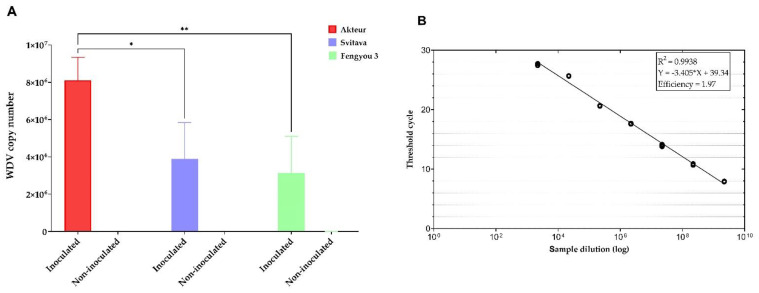
(**A**) The virus copy number detected in each individual wheat genotype by qPCR, two-way ANOVA and Tukey’s multiple comparison test was applied to detect the statistical significance of the WDV titre in inoculated Fengyou 3 and Svitava compared to Akteur (a susceptible genotype) where * = *p* < 0.05 and ** = *p* < 0.01. (**B**) The calibration curve for SYBR Green I-based qPCR amplification of standard WDV DNA, with the specific set of primers UnivWDVfw and UnivWDVrv [35] calculated based on the WDV copy number in each sample.

We used qPCR for absolute quantification of WDV in three wheat genotypes (Akteur, Svitava and Fengyou 3). WDV was detected in all inoculated plants but not in the control plants. The highest titre was obtained in the susceptible genotype Akteur (8.12 × 10^6^ copies), which was twice and thrice as high as in the Svitava genotype (3.89 × 10^6^ copies) and thrice as high as in the Fengyou 3 genotype (3.14 × 10^6^ copies), respectively (Figure 2, Supplementary Table S2). A two-way analysis (ANOVA) was conducted to analyse the effects of WDV inoculation and genotype on the WDV copy number. The analysis showed that there was a statistically significant interaction between the effects of virus inoculation and genotype (F (2, 13) = 6.52, *p* = 0.011). Both virus inoculation (*p* < 0.0001) and wheat genotype (*p* = 0.011) had statistically significant effects on virus copy numbers. Tukey’s test for multiple comparisons was applied to determine the statistical significance of WDV titre in inoculated Fengyou 3 and Svitava compared to Akteur (a susceptible genotype), with *p* < 0.05 and *p* < 0.01, respectively. The lower virus titres in the WDV-inoculated Fengyou 3 and Svitava genotypes compared to the Akteur genotype confirmed the higher levels of resistance to WDV observed in the form of milder disease symptoms in these genotypes (Figure 1).

### 3.2. Sequencing Output and Assembly

RNA from 72 samples corresponding to three *T. aestivum* genotypes (Akteur, Fengyou 3 and Svitava), two treatments (non-inoculated and infected with WDV) and four biological replicates were used for Illumina Genome Analyzer deep sequencing. At least 6.9 million raw reads were generated for non-inoculated and WDV-infected genotypes of *T. aestivum*. After cleaning, the number of reads was reduced to 6.8 million. In total, we generated 616 million raw reads and 604 million cleaned reads (Supplementary Table S3). The 72 sets of cleaned reads were mapped to the current reference wheat genome (IWGSC) using Bowtie2 v2.4.4 software (http://bowtie-bio.sourceforge.net/bowtie2/index.shtml, accessed on 5 March 2021). Approximately 259 (non-inoculated genotypes) and 234 million (WDV-infected genotypes) reads were mapped to the reference genome, accounting for 82.63% and 80.88% of the total clean reads for non-inoculated and WDV-infected genotypes, respectively (Supplementary Table S3).

### 3.3. Differential Expression Analysis

**Figure 3:**
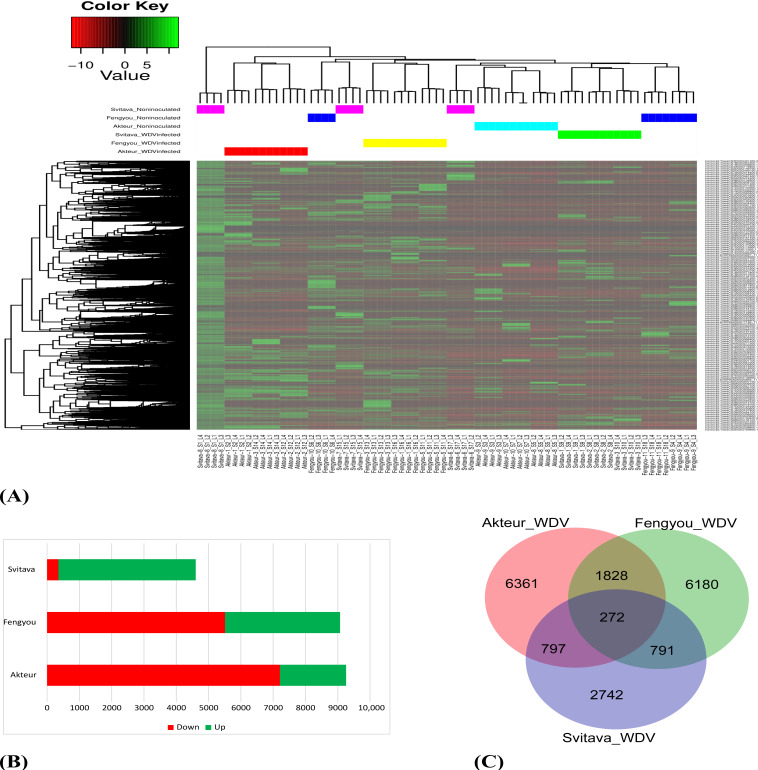
Summary of differential expression in WDV-infected and non-inoculated genotypes. (**A**) Heatmap of transcript expression and hierarchical clustering analysis of the WDV-infected and non-inoculated genotypes. (**B**) A bar chart showing the number of differentially expressed transcripts (DETs) in the three genotypes after WDV infection. (**C**) A Venn diagram showing the overlaps of the DETs of the three genotypes after WDV infection.

Transcript read counts were quantified based on fragments per kilobase per million (FPKM) normalised matrix to facilitate comparison of mRNA levels. Differentially expressed transcripts (DETs) (*p*-value < 0.001 and log2 (fold change) > 2) were defined as transcripts that were significantly enriched or depleted in WDV-infected genotypes compared to that in control genotypes (Supplementary Table S4). Based on the log_10_ RPKM of the 72 samples, hierarchical clustering of DETs was performed to observe the overall pattern of gene expression (Figure 2A,B). After a systematic assessment of the expression profiles of the mRNAs, we found that a total of 22,931 DETs were present in both non-inoculated and WDV-infected libraries. Akteur, Fengyou 3 and Svitava each expressed 9258, 9071 and 4602 DETs, respectively. Moreover, the number of downregulated transcripts was 7208 (~77.6%), 5509 (~60.7%) and 352 (~7.7%), while the number of upregulated transcripts was 2050 (~22.1%), 3562 (~39.3%) and 4250 (~92.4%) for the Akteur, Fengyou 3 and Svitava genotypes, respectively (Figure 3C). These results show that the overall transcriptional expression profile is lower in the susceptible genotype (Akteur) than in the resistant genotype (Svitava) due to WDV infection. Nevertheless, the number of DETs was greater in the Akteur genotype than in the Svitava genotype. Strikingly, the expression profile of the resistant genotype Fengyou 3 was comparable to that of the susceptible genotype Akteur (Figure 3A–C). Moreover, we identified 6361 and 6180 genotype-specific DETs in the Akteur and Fengyou 3 genotypes, respectively; only 2742 Svitava-specific DETs were found (Figure 3D). Furthermore, the resistance genotypes (Fengyou 3 and Svitava) had 791 DETs in common, while all genotypes had 272 DETs in common.

**Figure 4:**
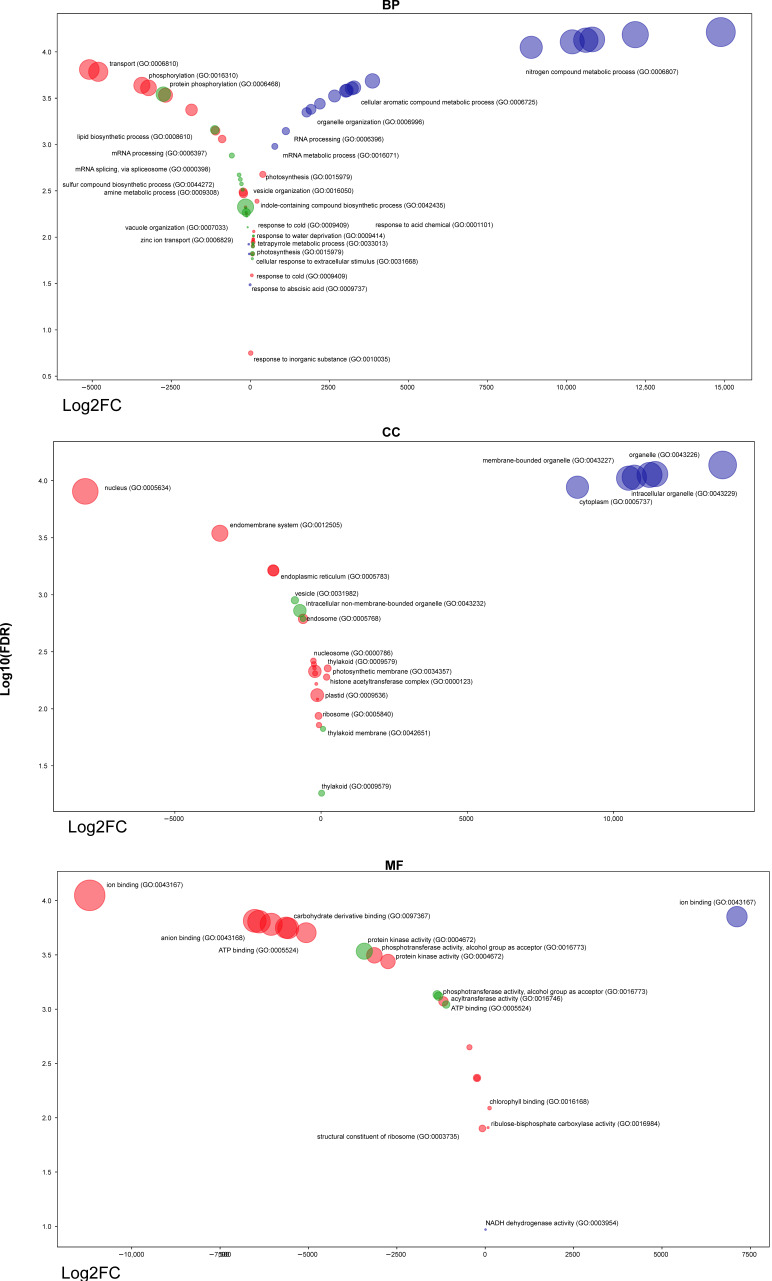
A bubble chart of annotated differentially expressed transcripts (DETs) and classification in the Gene Ontology (GO), with results grouped into three main categories: biological process (BP), cellular component (CC) and molecular function (MF). Bubbles were coloured (red, green and violet) according to the different genotypes (Akteur, Fengyou 3 and Svitava), respectively. Log2 FC = log2 fold of change of transcripts.

The PANTHER Classification System (v.14.0) was used for the functional statistical enrichment test for the DETs of the Akteur, Fengyou 3 and Svitava genotypes. Next, the obtained Gene Ontology (GO) categories were summarised using Revigo [48]. The result showed that a total of 114 GO terms were populated for the DETs of the Akteur, Fengyou 3 and Svitava genotypes. Of these, 64 (biological process), 28 (cellular component) and 22 GO terms (molecular function) were significantly enriched (*p* < 0.05) (Supplementary Table S5 and Figure 4).

A total of 8507 (~91.89%) of the 9258 DETs in the Akteur genotype were involved in differential binding activity in the molecular function category. The highest proportion of these DETs were involved in ion binding (GO:0043167), while the remaining DETs belonged to different subcategories such as phosphotransferase activity (GO:0016773), protein kinase activity (GO:0004672) and acyltransferase activity (GO:0016746) (Supplementary Table S5 and Figure 3). In lineage, DETs in the Fengyou 3 genotype were involved in binding activities such as small molecule binding (GO:0036094), but the majority were involved in protein kinase activity (GO:0004672). In addition, the majority of DETs in the Svitava genotype were involved in ion binding (GO:0043167) (Supplementary Table S5 and Figure 4). 

Furthermore, GO enrichment analysis showed that the predicted target genes were involved in a wide array of biological processes (Supplementary Table S5 and Figure 4). Among the DETs enriched in the Akteur genotype GO categories, localisation (GO:0051179), transport (GO:0006810), phosphorylation (GO:0016310), catabolic processes (GO:0009056) and mRNA processing (GO:0006397) were significantly repressed, while mRNA processing (GO:0006397), photosynthesis (GO:0015979), tetrapyrrole metabolic process (GO:0033013), carbon fixation (GO:0015977), response to cold (GO:0009409) and water deprivation (GO:0009414) were activated (Supplementary Table S5 and Figure 4). Similar to Akteur, the enriched GO categories of DETs in the Fengyou 3 genotype phosphorylation (GO:0016310) was suppressed and photosynthesis (GO:0015979) was activated. Interestingly, other biological processes such as lipid biosynthesis (GO:0008610), mRNA splicing (GO:0000398), amine metabolism (GO:0009308) and regulation of cellular ketone metabolism (GO:0010565) were suppressed, while cellular response to extracellular stimuli (GO:0031668) and zinc ion transport (GO:0006829) were activated. In contrast, the enriched GO categories of DETs in the Svitava genotype show significant activation of a wide range of biological processes, including cellular processes (GO:0009987), metabolic processes of nitrogen compounds (GO:0006807), organisation or biogenesis of cellular components (GO:0071840), nucleic acid metabolism (GO:0090304) and RNA processing (GO:0006396), while response to cold (GO:0009409) and response to water deprivation (GO:0009414) were suppressed (Supplementary Table S5 and Figure 4).

At the cellular component category, the enriched GO terms of the DETs in Akteur genotype corresponding to nucleus (GO:0005634) and endomembrane system (GO:0012505) were suppressed, while in Fengyou 3 the suppression of DETs was observed in the intracellular non-membrane-bounded organelle (GO:0043232), vesicle (GO:0031982) and endosome (GO:0005768). In contrast, the enriched GO terms of the DETs in Akteur genotype corresponding to almost all cellular components were activated (Supplementary Table S5 and Figure 4). 

**Figure 5:**
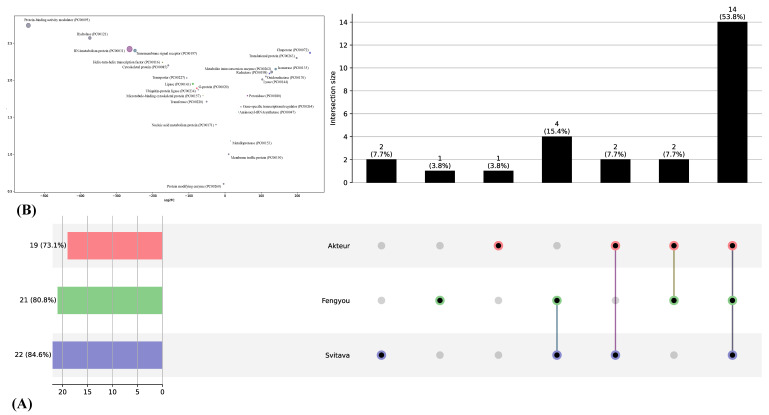
The functional significance of the shared and genotype specific DETs. (**A**) UpSet plot of the intersection among of the enriched PANTHER protein classes in the three genotypes. (**B**) A bubble plot of intersected enriched PANTHER protein classes expressions and gene ontology (GO) classification.

To gain insights into the functional significance of the common and genotype-specific DETs, we created UpSet plots of the enriched PANTHER protein classes for the three genotypes. The results showed that Akteur-specific DETs represent the repression of the protein class G (PC00020) (Figure 4). In addition, Fengyou 3-specific DETs represent the suppression of the protein class lipase (PC00143), while Svitava-specific DETs represent the activation of the protein classes chaperone (PC00072) and reductase (PC00198). Interestingly, resistant genotype-specific DETs represent activation of the protein classes isomerase (PC00135), metalloprotease (PC00153) and aminoacyl-tRNA synthetase (PC00047) and suppression of a transmembrane signalling receptor class (PC00197) (Figure 4). Finally, all genotypes under WDV infection showed activation of six protein classes, including lyase (PC00144) and oxidoreductase (PC00176), while eight protein classes were suppressed, including protein binding activity modulator (PC00095), transferase (PC00220), transporter (PC00227) and hydrolase (PC00121) (Figure 5).

### 3.4. Metabolic KEGG Pathway Analysis for DETs in the Tested Genotypes

Identified DETs were mapped to the KEGG database using the KEGG mapper tool to gain insight into the major metabolic pathways in response to WDV. Pathway reconstruction analysis assigned KEGG orthologous numbers to all DETs in each genotype and mapped them in all KEGG metabolic pathways. Table 1 shows 39 of them, representing the 14 global metabolic, stress- and immune response-related metabolic pathways groups. A summary of the inter-group analysis between resistant (Fengyou 3 and Svitava) and susceptible (Akteur) varieties revealed that the number of DETs in resistant genotypes (Fengyou 3 and Svitava) was lower than in susceptible genotype (Akteur) in all pathways except in 10, including the photosynthesis pathway. In line with the expression profiles results, DETs expressions of 36 (92.3%) pathways were downregulated in Akteur and Fengyou 3 genotypes, while DETs expression of all selected KEGG pathways in Svitava genotype was upregulated (Table 1).

The high number of downregulated DETs in the global metabolic pathways group in the susceptible (Akteur) (1296 DETs) than in the resistant (Fengyou 3) genotype (1156 DETs) suggests that WDV exploits the host metabolism during pathogenesis (Table 1). Surprisingly, the resistant Svitava genotype with a lower number of (597) DETs was able to reverse the expression of global metabolic pathways, suggesting a higher level of resistance to WDV, as shown in Figure 2. Similarly, and regardless of the number of DETs, the downregulation level of DETs mapped to the two signal transduction pathways (MAPK–plant and plant hormone) and metabolism of xenobiotics by cytochrome P450 were also higher in the susceptible (Akteur) than in the resistant (Fengyou 3) genotype, while the expressions were upregulated in Svitava genotype (Table 1). On the contrary, the downregulation level of DETs mapped to starch and sucrose metabolism were also lower in the susceptible (Akteur) than in the resistant (Fengyou 3) genotype (Table 1). Moreover, upregulation of the plant pathogen interaction and biosynthesis of secondary metabolites DETs in the Svitava genotype were assumed to implement a strong immune reaction against WDV. Overall, KEGG analysis of the DETs revealed pathways that are involved in immune responses of tested genotypes against WDV.

### 3.5. Transcription Factors in Relation to WDV Infection

For more insights, we identified differentially expressed transcripts with encoded transcription factors (TFs) and classified them based on the previously determined TFs families (Table S6). Our results showed that 1038 TFs belonging to 54 TF families were differentially expressed due to WDV infection (Supplementary Tables S5 and S6). Moreover, TFs families with a well-established contribution to the way plants react to abiotic stress such as basic helix–loop–helix (bHLH), basic leucine zipper (bZIP), Cys2/His2-type (C_2_H_2_), MYB, NAC (NAM, ATAF and CUC) and WRKY were identified with a high number (Supplementary Table S6). Interestingly, AP2/ERF family had the highest number (103 TFs) of the differentially expressed TFs.

### 3.6. Validation of the DETs by RT-qPCR Analysis

**Figure 6:**
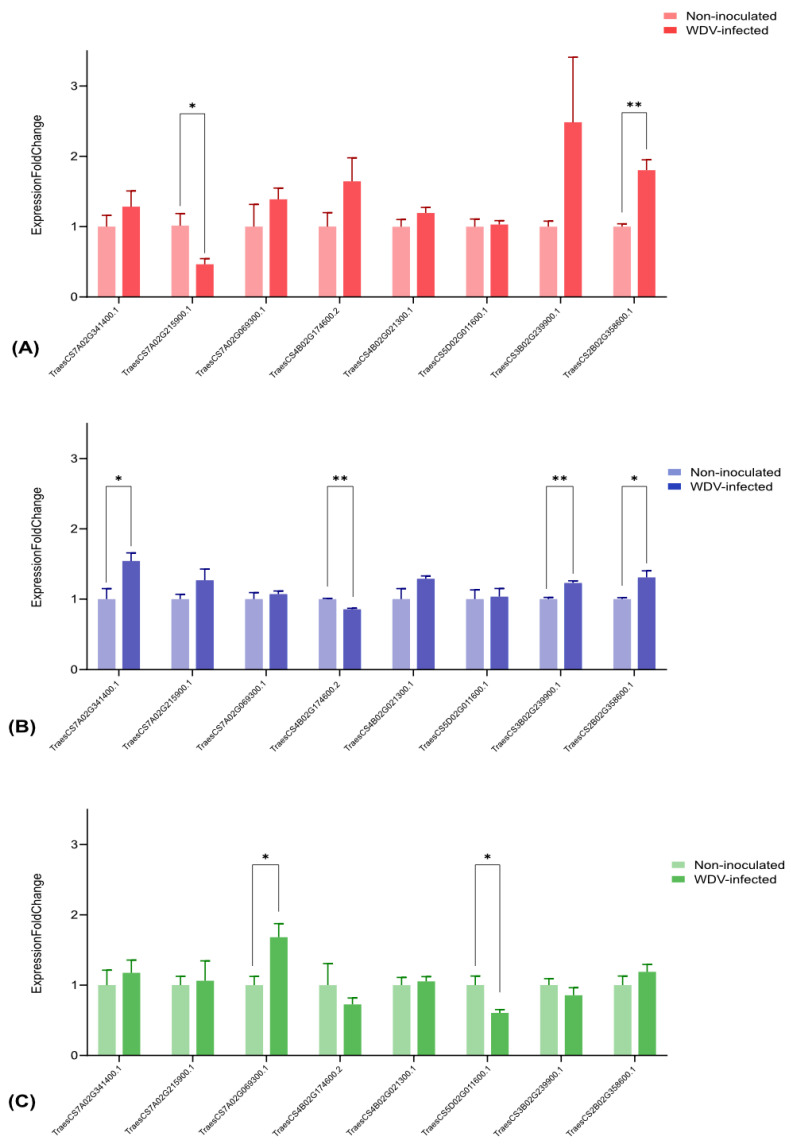
RT-qPCR analyses of eight WDV infection-related transcripts in WDV-infected and non-inoculated (**A**) Akteur, (**B**) Svitava and (**C**) Fengyou 3 genotypes. Each bar represents the mean ± SEM of triplicate assays. T Test, Holm-Šídák method (* = *p* < 0.05 and ** = *p* < 0.01).

To assess the reliability and validity of our transcriptome in the identified DETs, a total of eight DETs were selected and validated by RT-qPCR analysis (Supplementary Table S1). Figure 6 shows that all DETs were differentially expressed between WDV-infected and non-inoculated genotypes. The expression pattern of glycosyltransferase (TraesCS7A02G215900.1) was significantly downregulated by WDV infection in the susceptible genotype (Akteur) compared to the other genotypes (Fengyou 3 and Svitava), while CYCLIN-T1-3 (TraesCS2B02G358600), a regulator of cyclin-dependent kinases (CDK) was upregulated. On the other hand, the expression pattern of the transcription factor MYB (TraesCS4B02G174600.2) was downregulated by WDV infection in the resistant genotypes (Fengyou 3 and Svitava) compared to the susceptible genotype (Akteur), while two uncharacterised proteins (TraesCS7A02G341400.1 and TraesCS3B02G239900.1) involved in transport and regulation of cell growth, respectively, were upregulated. In general, the RT-qPCR results were comparable to the RNA-Seq-based gene expression patterns (Supplementary Table S4 and Figure 6).

## 4. Discussion

In this work, we investigated the transcriptome profiles changes of resistant (Svitava and Fengyou 3) and susceptible (Akteur) wheat genotypes to WDV. We confirmed the resistance or susceptibility of the genotypes by disease symptoms observation (Figure 1) and analysis of WDV titre by qPCR (Figure 2). WDV infection in resistance genotypes (Svitava or Fengyou 3) showed very mild disease symptoms and low virus titre in comparison to the susceptible genotype, Akteur, where severe disease symptoms including yellowing, dwarfing and high reduction of root system were observed as well as the high virus titre. High levels of plant resistance to viruses are usually characterised by mild disease symptoms and reduced virus replication [15,17,49,50]. A similar resistance phenotype Svitava [16] or Fengyou 3 was found in several winter wheat varieties (e.g., Mv Vekni, Mv Dalma, Banquet, Svitava, Fisht) [8,15,16,17]. Otherwise, most of the known genotypes and germplasm accessions or wild relatives of wheat have different levels of susceptibility to WDV, resulting in huge yield losses [8,11,17,51] and affecting various traits such as reduction in plant height and the root system [8,51]. Recently, however, some sources of WDV resistance have been found in genebank accessions, including di-, tetra- and hexaploid wheat, and members of quantitative trait loci (QTL) for partial resistance have been identified [17], which could improve wheat breeding for WDV resistance. The Svitava (Czech origin) and Fengyou 3 (Chinese origin) genotypes, which have showed high resistance to WDV and molecular analyses such as the transcriptome, have made it possible to identify putative new genes associated with resistance to the virus. Our RNA-Seq analysis showed that 9258, 9071 and 4602 transcripts were significantly differentially expressed in response of WDV infection in the Akteur, Fengyou 3 and Svitava genotypes, respectively (Figure 3). This finding reveals specificity of transcript expression profile associated with resistance (Svitava, Fengyou 3) and susceptible (Akteur) genotypes. We found that out of a total 114 significantly enriched GO terms, 64 biological process, 28 cellular components and 22 belonged to the molecular function in the tested genotypes (e.g., Akteur, Fengyou 3 and Svitava) (Supplementary Table S5 and Figure 4). In general, the resistant genotype showed significant activation of biological processes after WDV infection compared to the susceptible genotype (Figure 4 and Figure 5). The number of genes were distinctly upregulated and downregulated based on the level of resistance to WDV (Figure 6, Table 1). Altogether, the binding and transporting activity was negatively affected due to the WDV infection, while oxidoreductase (PC00176) and lyase (PC00144) were activated (Figure 4). The suppression of the transporting activity might be the plant’s response to prevent virus intracellular movement from the replication site to plasmodesmata, reducing virus spread [52]. Moreover, suppression of the transporting activity might be the plant’s response to prevent virus accumulation. Recent reviews show that plant antiviral defences restrict essential parts of the infection cycle, such as viral RNA translation, virus replication or movement, resulting in reduced and/or delayed establishment of systemic infection [53,54]. On the other hand, the activation of oxidoreductase (PC00176) is expected as it is an enzyme that catalyses a reduction–oxidation (redox) reaction (Figure 4). Activation of redox status is one of the earliest responses detected as a plant immune response [55]. Previous results prove that lyases triggered defence plant responses by limiting virus accumulation and moderating symptom severity [56]. Interestingly, the resistance genotypes have different pathways to cope with virus infection. The Svitava genotype effectively copes with virus infection by suppressing chaperones (PC00072) which includes heat shock protein (HSP) families (Figure 3). These cytoplasmic proteins are transformed into an aggregated state in infected plant cells, which together with protein quality control and autophagy proteins play a major role in viral DNA/proteins and virion aggregation and, consequently, in virus mobilisation [57,58,59]. Furthermore, HSPs play a role in decreasing viral amounts when HSP70 is inhibited [60]. Moreover, the HSP families have endoplasmic reticulum (ER)-associated protein folding. The ER stress produces the unfolded protein response when plants are subjected to severe or chronic stress, which boosts programmed cell death [61,62]. Surprisingly, the Svitava genotype specifically-suppresses the reductase (PC00198) protein class (Figure 4). Reductase is a well-known enzyme involved in the detoxification of reactive oxygen species (ROS). Recently, plants with less ROS accumulation show better adaptation to virus infections and are linked to salicylic acid signal deficiency [63]. In agreement with previous reports, the expression patterns of cell wall- and chloroplast-related genes changed significantly in response to virus infection [25,40], including WDV [64]. 

In this study, both GO and KEGG pathway analyses emphasised a post-inoculation re-programming of the several regulated transcripts, revealing that the different responses to WDV infection between genotypes were based on many pathways such as carbohydrate, energy, lipid, nucleotide, amino acid, glycan and vitamins metabolism. The differences in gene expression following WDV infection might likely have contributed to different resistance levels of these three genotypes. The transcripts in both secondary metabolism and metabolism pathways were induced in the highly resistant genotype (Svitava) (Table 1). Similar induction was found in several plant–virus interactions [64,65,66,67,68,69]. Genes of carbon fixation in photosynthetic pathways (map00710) were repressed in the susceptible genotype (Akteur), while they were induced in the resistant genotypes (Fengyou 3 and Svitava) (Table 1). This result is consistent with the severe leaf chlorosis observed in the Akteur genotype compared to Fengyou 3 and Svitava (Figure 1). Chloroplast acts as a defence signal generator and stress sensor; besides which, it has a principal metabolic energy function [70,71]. The chlorosis region is usually associated with virus accumulation and clustering [65,70,71,72]. Several reports on plant–virus interactions showed the downregulation of photosynthesis and chloroplast-related genes associated with infection symptoms development [63,65,72,73,74]. Moreover, the virus can affect chloroplast ultrastructure and interact directly with chloroplast components [74,75,76,77]. Furthermore, the role of chloroplast in the replication and movement of plant viruses and plant defence against viruses has been reported [63,72,75]. However, the virus mechanism to downregulate photosynthesis-related genes remains unclear. Our study suggests the photosystem’s important role in WDV infection. The biosynthesis of other secondary metabolites such as flavonoid (biosynthesis: map00941) transcripts are upregulated in the Svitava genotype and downregulated both in Fengyou 3 and Akteur, which are presumably associated with higher levels of resistance to WDV that are perhaps due to their antiviral properties through inhibiting viral polymerases and binding to the nucleic acids or capsid proteins of the virus [78]. Similarly, GETs related to plant–pathogen interaction (map04626) are highly enriched in Svitava, which is in accordance with resistant patterns and in agreement with the findings of recent studies of viruses from distinct hosts [31,69]. The signal transduction pathways, in particular the MAPK (map04016) and plant hormone signal transduction (map04075), with associated GETs are highly enriched in resistance genotype, which again suggests their key role in gene regulation conferring plant resistance to viruses [31,32,69]. 

Transcription factors (TFs) control gene expression by attaching to genes’ cis-acting elements. TFs initiate the signal transduction networks of stress signals (Supplementary Table S5). Moreover, several TFs that respond to biotic and abiotic stresses have been reported [79,80], suggesting that they may be necessary for the adaptation to different environments. Many TFs families such as AP2/ERF, bHLH, NAC, WRKY and MYB include TFs that control different stresses in plants. In our study, TFs of AP2/ERF, bHLH, MYB and WRKY families were highly enriched under WDV infection (Supplementary Table S5). The bHLH TFs play a role in plant response to stress and are involved in plant photomorphogenesis, light signal transduction and secondary metabolism [81]. The MYB TFs have a key function in plant hormone synthesis, signal transduction and plant responses to various biotic and abiotic stresses [82]. WRKY is one of the largest TF families and has a role in abiotic and biotic stresses by regulating the signal transduction pathway of plant hormones [83]. All three TFs families are well characterised and their role concerning abiotic stress in plants was massively investigated while reports about abiotic stress particularly virus infection have been less abundant [83,84]. Interestingly, ERF is the highest TFs family identified in our study and is widely reported to be involved in responses to numerous biotic stresses in diverse plant species [85]. ERFs are part of the (AP2/ERFs) TF family and are one of the biggest plant TF families. ERFs regulate ethylene signalling and play roles in plant resistance to pathogens and fruit ripening [85]. Recently, ERF association with plant immune responses and resistance against plant viruses has been reported and its virus resistance mechanism is under investigation [86,87].

## 5. Conclusions

RNA sequencing reveals comprehensive genome-wide expression profiles of virus-responsive transcripts in resistant and susceptible wheat genotypes that enabled us to investigate the molecular determinants conferring WDV-resistance. The resistant Svitava genotype displayed a prompt and boosted immune reaction and responded to WDV by provoking the expression of pathogen-responsive transcripts that suppressed the viral infection process. For the first time, we investigated genome-wide expression profiles of bread wheat genotypes with different levels of host–virus interaction, which would assist in the identification of transcripts that could be employed for designing plant-targeted control strategies against the viral pathogen. In line with previous reports, our results show that WDV infection suppresses the binding and transporting activity while oxidoreductase and lyase were triggered in all three genotypes. Moreover, cell wall- and chloroplast-related transcripts were differentially expressed. Based on GO and KEGG analyses, DETs implicated in defence response, such as plant–pathogen interaction, biosynthesis of secondary metabolites, signal transduction pathways (MAPK-plant and plant hormone) and metabolism of xenobiotics pathways, were all related to the resistant background that is functionally involved in pathways to biotic stresses, defence signalling, transcriptional regulation, antioxidant activity and biosynthesis of secondary metabolites. Our findings suggest that resistance genotypes cope with virus infection through different pathways. Svitava in particular effectively copes with infection by repressing HSPs and reductase protein classes.

## Figures and Tables

**Table 1:** List of top metabolic pathways as revealed by KEGG enrichment analysis, showing the number of DTEs that were mapped to different KEGG pathways and its collective log2 fold change (Log2FC).

Metabolism	KEGG Pathways (KEGG Map)	Wheat Genotype					
		Akteur ***		Fengyou 3 *		Svitava *	
		Number of DETs	FDR	Number of DETs	FDR	Number of DETs	FDR
Global metabolism	Carbon metabolism (map01200)	146	−24.746	96	−25.893	64	418.701
	Biosynthesis of amino acids (map01230)	129	−35.222	105	−66.673	57	445.971
	Biosynthesis of secondary metabolites (map01110)	791	−364.09	729	−902.12	334	2259.25
	Metabolic pathways (map01100)	1296	−686.99	1156	−809.86	597	4090.61
Carbohydrate metabolism	Glycolysis/Gluconeogenesis (map00010)	62	−76.394	52	−87.632	34	206.022
	Starch and sucrose metabolism (map00500)	75	−15.253	70	−05.103	32	237.417
	Glyoxylate and dicarboxylate metabolism (map00630)	38	49.985	24	−9.2318	15	62.9341
	Fructose and mannose metabolism (map00051)	33	−59.557	15	5.72201	14	85.7023
	Ascorbate and aldarate metabolism (map00053)	23	−32.463	25	−01.951	16	133.646
	Pentose phosphate pathway (map00030)	28	−3.8213	19	−3.3531	13	69.6349
	Amino sugar and nucleotide sugar metabolism (map00520)	63	−78.453	51	−80.561	35	194.476
Energy metabolism	Oxidative phosphorylation (map00190)	50	−77.127	38	−7.5956	37	160.061
	Photosynthesis (map00195)	46	14.5403	20	72.2376	21	103.926
	Carbon fixation in photosynthetic organisms (map00710)	59	−3.6382	27	43.2827	21	112.541
	Nitrogen metabolism (map00910)	26	−33.916	17	−8.3395	4	23.2274
Lipid metabolism	Fatty acid biosynthesis (map00061)	11	−1.2844	24	−37.331	12	98.2974
	Glycerolipid metabolism (map00561)	39	−26.269	37	−85.768	20	104.584
	Sphingolipid metabolism (map00600)	22	−98.815	23	−155.87	14	125.386
	Glycerophospholipid metabolism (map00564)	51	−95.534	57	−223.72	22	115.207
Nucleotide metabolism	Purine metabolism (map00230)	41	−17.537	52	−68.903	26	182.311
	Pyrimidine metabolism (map00240)	38	−37.685	33	−6.5075	14	119.248
Amino acid metabolism	Cysteine and methionine metabolism (map00270)	49	−71.645	51	−34.552	37	300.873
	Arginine biosynthesis (map00220)	24	−42.567	15	−6.6615	8	63.7785
	Phenylalanine, tyrosine and tryptophan biosynthesis (map00400)	29	−81.181	34	−64.583	16	126.97
	Alanine, aspartate and glutamate metabolism (map00250)	35	−03.947	19	−3.0665	14	77.4047
	Glutathione metabolism (map00480)	61	−66.911	62	−22.133	30	209.633
Glycan biosynthesis and metabolism	N-Glycan biosynthesis (map00510)	25	−49.812	25	−30.927	15	108.616
Metabolism of cofactors and vitamins	Vitamin B6 metabolism (map00750)	12	−7.7555	12	−0.16818	11	66.7719
	Folate biosynthesis (map00790)	11	−4.7568	21	−37.222	12	115.05
Metabolism of terpenoids and polyketides	Terpenoid backbone biosynthesis (map00900)	36	−25.709	22	−1.6716	14	126.986
	Diterpenoid biosynthesis; Including: Gibberellin biosynthesis (map00904)	15	−5.7805	10	−02.006	2	15.434
	Carotenoid biosynthesis (map00906)	19	−1.9556	23	−3.7998	4	1.20272
Biosynthesis of other secondary metabolites	Flavonoid biosynthesis (map00941)	46	−263.47	37	−55.129	8	45.6125
Environmental adaptation	Plant-pathogen interaction (map04626)	109	−47.313	119	−34.729	60	459.918
Folding, sorting and degradation	Protein processing in endoplasmic reticulum (map04141)	123	−81.442	103	−01.685	43	355.089
Signal transduction	Mitogen-activated protein kinase (MAPK) signalling pathway—plant (map04016)	96	−74.825	91	−26.981	39	244.76
	Plant hormone signal transduction (map04075)	100	−58.428	117	−36.569	52	396.553
Xenobiotics biodegradation and metabolism	Metabolism of xenobiotics by cytochrome P450 (map00980)	65	−431.97	73	−48.641	21	134.894

*** Susceptible; * Highly resistant.

## Data Availability

The 72 mRNA-sequencing datasets are available at the NCBI Gene Expression Omnibus (GEO) with accession no. GSE192518.

## Supplementary Materials

The following supporting information can be downloaded at: https://www.mdpi.com/article/10.3390/v15030689/s1, Table S1. Primers for RT-qPCR analysis; Table S2. virus copy number detected in each individual wheat genotype by qPCR; Table S3 summary of the sequencing results; Table S4 differentially expressed transcripts in the three genotypes under the WDV infection; Table S5 summary of the annotated differentially expressed transcripts (DETs) expressions and gene ontology (GO) classification, with the results summarised in three main categories: biological process (BP), cellular component (CC) and molecular function (MF); Table S6. transcription factors in relation to WDV infection.
